# One-stop-shop cardiac CT: Calcium score, angiography, and myocardial perfusion

**DOI:** 10.1007/s12350-015-0351-9

**Published:** 2015-12-11

**Authors:** Alexander R. van Rosendael, Aukelien C. Dimitriu-Leen, Jeroen J. Bax, Lucia J. Kroft, Arthur J. H. A. Scholte

**Affiliations:** 1 Department of Cardiology, Leiden University Medical Center, Leiden, The Netherlands; 2 The Interuniversity Cardiology Institute of the Netherlands, Utrecht, The Netherlands; 3 Department of Radiology, Leiden University Medical Center, Leiden, The Netherlands

## Case

**Figure 1:**
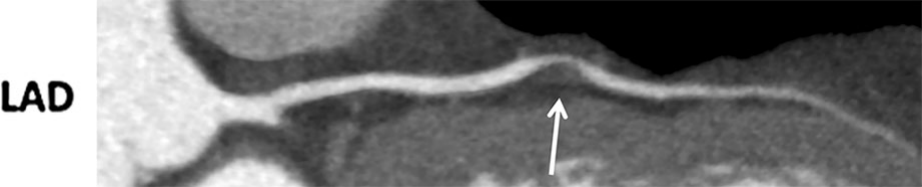
Multiplanar reconstruction of the left anterior descending artery (LAD) demonstrating a severe non-calcified lesion in the mid-LAD

**Figure 2:**
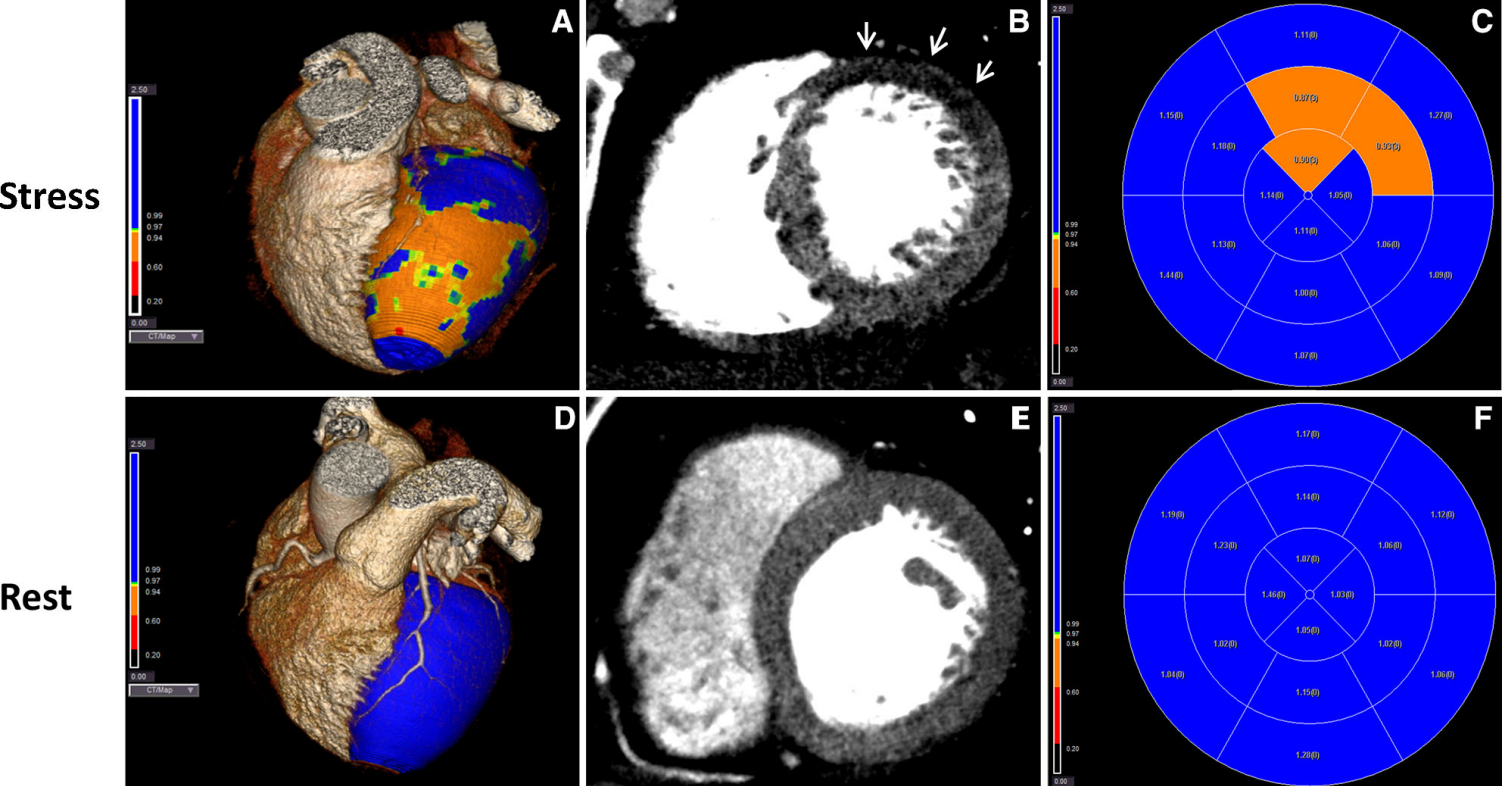
Images of CTP during stress (**A**-**C**) and rest (**D**-**F**). During adenosine stress, 3D fusion (**A**), short-axis reconstruction (**B**) and polar plot display (**C**) demonstrate anterolateral hypo-enhancement. (**D**-**F**) Rest CTP, with same reconstructions as stress, demonstrate normal myocardial enhancement

**Figure 3:**
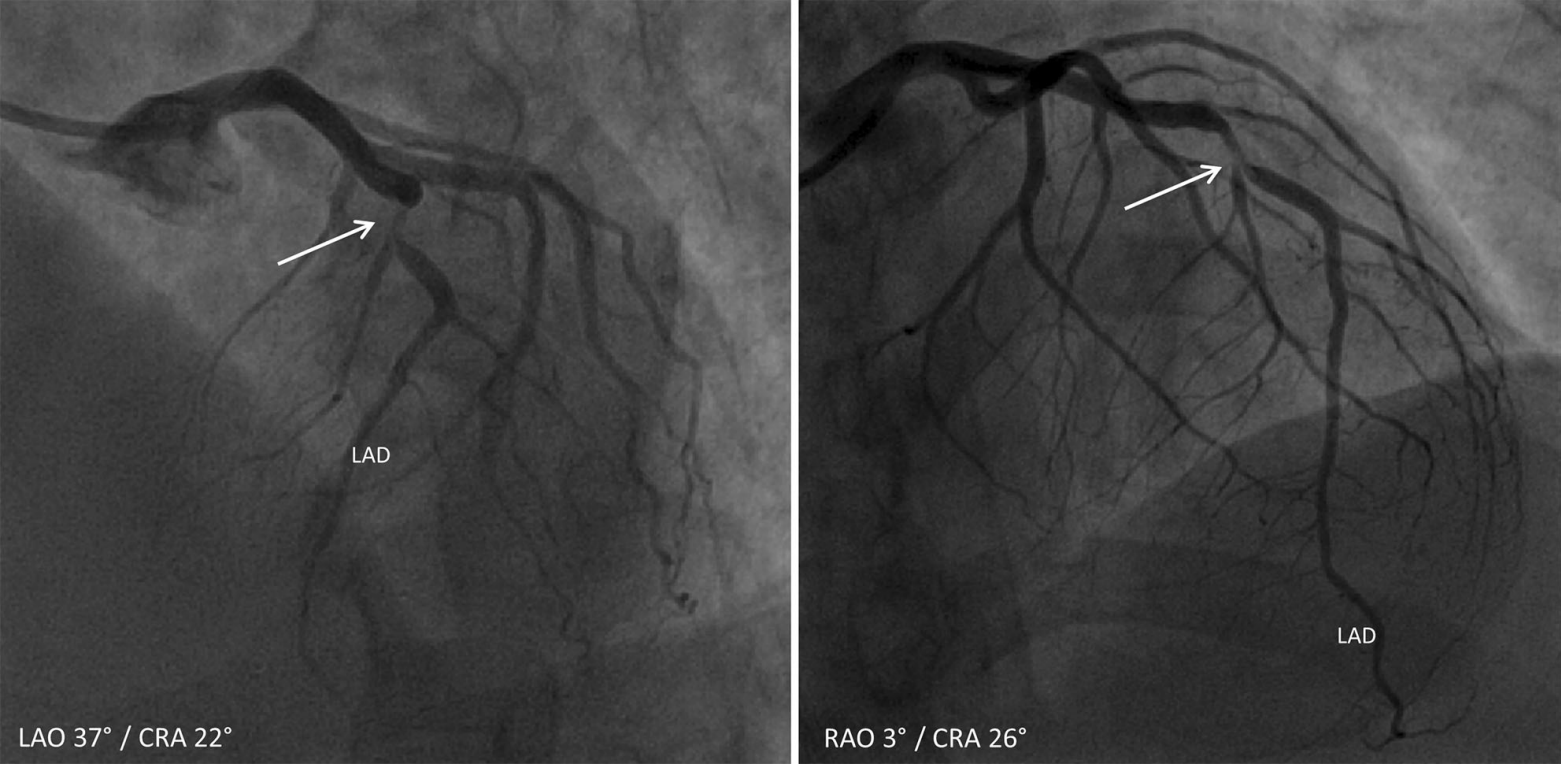
Invasive coronary angiography demonstrating a significant lesion in the mid left anterior descending artery

**Figure 4:**
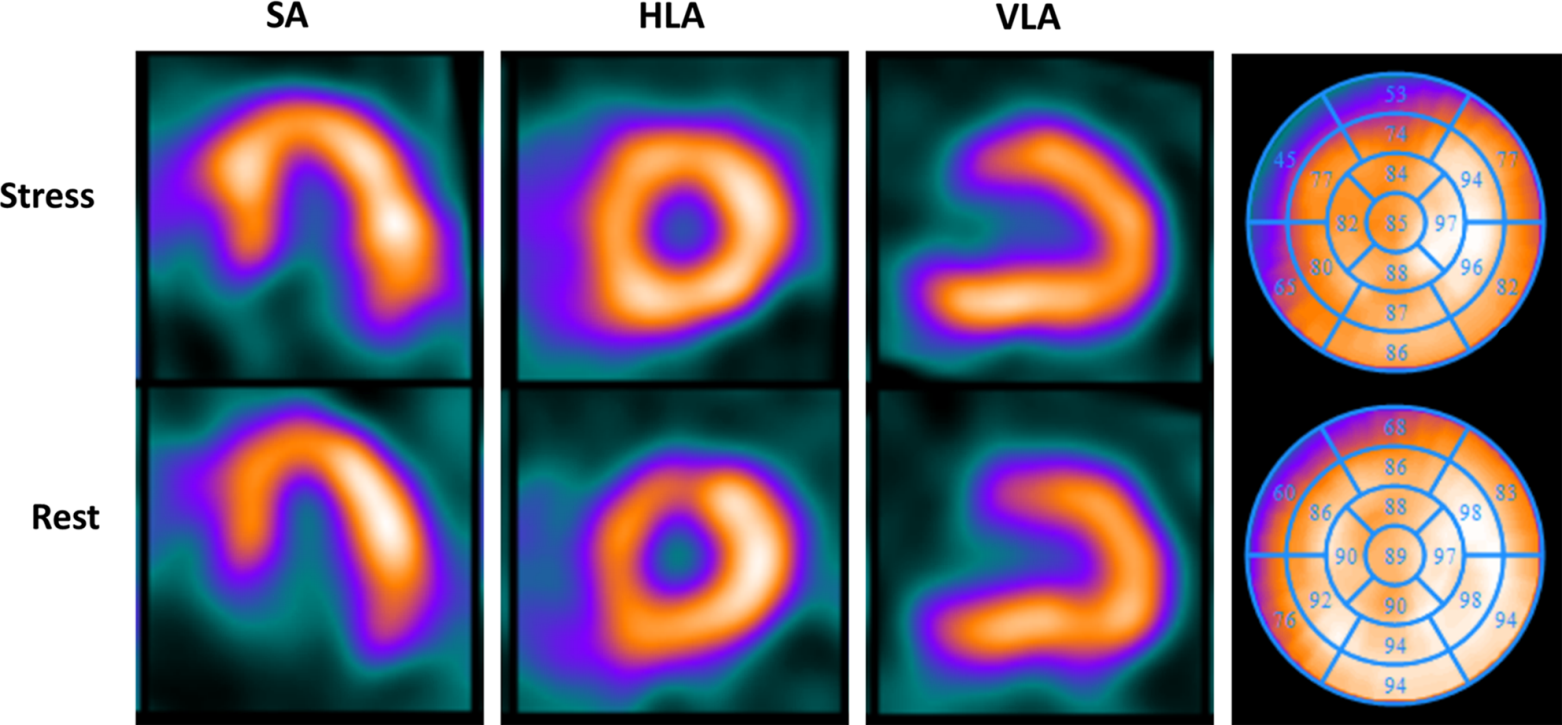
SPECT MPI images in short-axis (SA), horizontal long-axis (HLA), vertical long-axis (VLA), and polar plot demonstrating no perfusion defect at stress and rest

A 52-year-old man presented to the outpatient clinic with dyspnea on exertion and atypical angina. Cardiovascular risk factors were a history of smoking (25 pack years) and hypertension, and the pretest risk probability for coronary artery disease was 49%.^1^ The coronary artery calcium score (CACS) was 0; however, because of symptoms coronary CT angiography (CTA) was performed which showed a severe non-calcified lesion in the mid left anterior descending artery (LAD), Figure 1. Sequentially, an adenosine stress CT myocardial perfusion (CTP) was performed showing a reversible, anterolateral (>50% transmural) perfusion defect, Figure 2B. Invasive coronary angiography confirmed the significant stenosis in the mid-LAD (Figure 3), which was stented. Due to persisting symptoms single-photon emission computed tomography (SPECT) myocardial perfusion imaging (MPI) was performed, which showed dissolved ischemia anterolateral without new perfusion abnormalities, Figure 4.

## Discussion

In patients with symptoms and a CACS of 0, coronary CTA should be performed to rule out significant CAD.^2^ However, if obstructive CAD is observed, hemodynamic consequences need to be further evaluated to decide whether treatment with revascularization is needed. The possibility to perform CTP imaging in the same setting is an efficient way to diagnose hemodynamically significant CAD; the so-called one-stop-shop!
